# Comparing between Different Sets of Preprocessing, Classifiers, and Channels Selection Techniques to Optimise Motor Imagery Pattern Classification System from EEG Pattern Recognition

**DOI:** 10.3390/brainsci12010057

**Published:** 2021-12-31

**Authors:** Francesco Ferracuti, Sabrina Iarlori, Zahra Mansour, Andrea Monteriù, Camillo Porcaro

**Affiliations:** 1 Department of Information Engineering, Università Politecnica delle Marche, 60131 Ancona, Italy; f.ferracuti@univpm.it (F.F.); s.iarlori@univpm.it (S.I.); zahramansour94@gmail.com (Z.M.); a.monteriu@univpm.it (A.M.); 2 Department of Neuroscience and Padova Neuroscience Center (PNC), University of Padova, 35128 Padova, Italy; 3 Institute of Cognitive Sciences and Technologies (ISCT)—National Research Council (CNR), 00185 Rome, Italy; 4 Centre for Human Brain Health, School of Psychology, University of Birmingham, Birmingham B15 2TT, UK

**Keywords:** brain-computer interface (BCI), electroencephalography (EEG), Support Vector Machine (SVM), K-Nearest Neighbors (KNN), decision tree, imagination movement (IM)

## Abstract

The ability to control external devices through thought is increasingly becoming a reality. Human beings can use the electrical signals of their brain to interact or change the surrounding environment and more. The development of this technology called brain-computer interface (BCI) will increasingly allow people with motor disabilities to communicate or use assistive devices to walk, manipulate objects and communicate. Using data from the PhysioNet database, this study implemented a pattern classification system for use in a BCI on 109 healthy volunteers during real movement activities and motor imagery recorded by 64-channels electroencephalography (EEG) system. Different classifiers such as Support Vector Machine (SVM), K-Nearest Neighbors (KNN), and Decision Trees (TREE) were applied on different combinations of EEG channels. Starting from two channels (C3, C4 and CP3 and CP4) positioned on the contralateral and ipsilateral sensorimotor cortex, the Region of Interest (RoI) centred on C3/Cp3 and C4/Cp4 and, finally, a data-driven automatic channels selection was tested to explore the best channel combination able to increase the classification accuracy. The results showed that the proposed automatic channels selection was able to significantly improve the performance of each classifier achieving 98% of accuracy for classification of real and imagined hand movement (sensitivity = 97%, specificity = 99%, AUC = 0.99) by SVM. While the accuracy of the classification between the imagery of hand and foot movements was 91% (sensitivity = 87%, specificity = 86%, AUC = 0.93) also with SVM. In the proposed approach, the data-driven automatic channels selection outperforms classical a priori channel selection models such as C3/C4, Cp3/Cp4, or RoIs around those channels with the utmost accuracy to help remove the boundaries of human communication and improve the quality of life of people with disabilities.

## 1. Introduction

The number of people who suffer from temporal or permanent movement disabilities is enormously growing worldwide. It is estimated to range between 20–50 million people depending on the World Health Organization (WHO) [1]. People with such motor deficiencies faced an encumbrance on performing their daily activities [2]. Therefore, many intelligent assistive systems have been developed to improve the Quality of Life (QoL) for people with movement disabilities by facilitating communication and creating a smart interactive environment [3]. One of the significant intelligent assistive systems depends on acquiring and transforming the activation of the Human Central Nervous System (CNS) into an artificial output that can be controlling an external device or lead a prosthesis: such a system is called Brain-Computer Interface (BCI) [4].

The research on BCI started in 1973 [5] when a group of scientists at the University of California proposed for the first time the BCI expression and launched the BCI challenge, which aims to control an external object using the brain signal recorded by Electroencephalography (EEG) [6]. After BCI was defined by Vidal [5], the first use of a frequency band in a BCI was presented in 1988 [7,8]. Later papers used beta and other bands. The 1988 work on BCI robot control explicitly uses [9] an artificial intelligence (AI) algorithm with machine learning (training) period and examination (testing) period. The development of the AI algorithms provided a learning pattern recognition approach [10], helping to increase the quantity and the quality of the BCI research in general and, in particular, on the Motor Imagery (MI). In this paradigm, the visuomotor imagery has been used to replace the real execution of the movement [11]. MI is a voluntarily generated signal and is dependent on the subject’s intention that represents one of the most common paradigms because it can be used with stroke patients who suffer from movement disabilities. At the same time, their mental imagery can work properly [12].

Regardless of the application, the first step of any BCI system is the acquiring of the brain activity usually performed by the EEG system thanks to their portability, cost less, and its high temporal resolution, and non-invasivity [13,14,15]. On the other hand, its non-invasivity decreases the quality of the acquired signals that are corrupted by biological and non-biological artefacts [16,17,18]. Thereover, different preprocessing techniques have been proposed to increase the EEG signal-to-noise ratio (SNR) such as Independent Component Analysis (ICA) based algorithm both online [19,20,21] and offline [16,18]. However, this step is very time-consuming and ICA experts are needed to perform it. Thus, recently semi- and full-automated systems have been developed to eliminate those artefacts from EEG recordings such as SASICA, namely Semi-Automatic Selection of Independent Components for Artifact [22].

The vital core for any pattern classification system used in a BCI is represented by translating the cleaned signal into a matrix of features. This can be carried out by investigating the EEG signal in the time domain and using a combination of EEG signals from all channels and calculating different values such as amplitude measurements [23], while other techniques are based on Power Spectrum Density (PSD) analysis on some specific frequency bands [24].

One of the most used feature in motor execution (ME) and MI is the Event-Related Desynchronisation and Synchronisation (ERD/ERS) [25,26,27] in the alpha band [8–13 Hz] and the beta bands [14–25 Hz] in the contralateral and ipsilateral sensorimotor areas [28,29]. ERD and ERS were introduced by Pfurtscheller in 1999 [27]. The ERD/ERS patterns showed high suitability in a pattern classification system implemented in a BCI, especially in the discrimination between right and left-hand movement. Moreover, they gave accuracy higher than 80% for online classification [30].

High-density EEG (hdEEG), on the one hand, gives higher spatial accuracy of the signal recorded [19], but, on the other hand, the large number of EEG channels presents a significant challenge in developing any BCI system [31]. Therefore, selecting the optimal channels combinations is one of the most active research fields because it has a massive effect on the computational load in pattern classification system performance and avoiding overfitting [32].

According to other papers as [33] that presents a new method for EEG channel selection optimisation based on relief function [34], the proposed approach aims to realise a data-driven procedure, considering every feature highly affected by channel selection and avoiding calculating the summation of features weight of each channel as in [33], in order to increase the accuracy performance.

In this study, we aim to create a pattern classification system for use in a BCI to classify between the real and imagined movement of the hands and foot on data from 109 volunteers taken from PhysioNet database [35] by computing and comparing pre-processed data throughout a semi-automatic ICA (saICA) [16,18] full automated ICA (SASICA) [22] and only filtered data. ERD features in different frequency bands, alpha [8–13 Hz], beta [14–25 Hz], and alpha plus beta [8–25 Hz] were extracted and different channel configurations, C3 and C4, Region of Interest (RoI) around C3 and C4, and optimised algorithm methods for channel selection were compared as well as different classifiers such as Support Vector Machine (SVM), K-Nearest Neighbors (KNN), and Decision Trees (TREE).

## 2. Materials and Methods

The data used in this work were obtained from the EEG Motor Movement/Imagery Dataset V 1.0.0 [35]. This dataset consists of EEG recordings from 109 participants involving 4 tasks and 14 experimental runs. Nine subjects were excluded from the database (subject Number: 38, 88, 89, 92, 93, 94, 100, 104 and 106) because their data contains incorrectly annotated errors labels. The participants performed the MI tasks, while a 64-channel EEG signal was recorded with a BCI2000 system [31] using the international 10–10 system with a 160 Hz sampling rate and an average reference. The MI is induced by a target that appears in one of four possible locations along the right edge of the screen. Then, a cursor appears at the left edge and moves from left to right at a constant rate with its vertical movement controlled by the power in an α or β band at a location over sensorimotor cortex.

The participants performed 14 experimental runs: 2 baseline runs (1 with eyes open, ‘run 1’, and 1 with eyes closed, ‘run 2’, of 1 min each), and 3 runs for each of the following 4 task combinations (2 min each):

(i) Experimental runs 3, 7, 11. A target appears on either the left or the right side of the screen. The participant opens and closes the corresponding fist until the target disappears (Real Hand Movement “RHM”). Then the participant relaxes.
(ii) Experimental runs 4, 8, 12. A target appears on either the left or the right side of the screen. The participant imagines opening and closing the corresponding fist until the target disappears (Imagery Hand Movement “IHM”). Then the participant relaxes.
- Experimental runs 5, 9, 13. A target appears on either the top or the bottom of the screen. Next, the participant opens and closes either fists (if the target is on top) and both feet (if the target is on the bottom) until the target disappears (Real Fists or Feet Movement “RFM”). Then the participant relaxes.
(iv) Experimental runs 6, 10, 14. A target appears on either the top or the bottom of the screen. The participant imagines opening and closing either both fists (if the target is on top) or both feet (if the target is on the bottom) until the target disappears (Imagery Fists or Feet Movement “IFM”). Then the participant relaxes.

In this study, we have studied four different combinations of all the tasks despite to concentrate only on the motor imagery movement as other papers did [36,37] as follow:

(i) Real Hand Movement (RHM) vs. Imagery Hand Movement (IHM): RHM vs. IHM;
(ii) Real Fists or Feet Movement (RFM) vs. Imagery Fists or Feet Movement (IFM): RFM vs. IFM;
(iii) Real Hand Movement (RHM) vs. Real Fists or Feet Movement (RFM): RHM vs. RFM;
(iv) Imagery Hand Movement (IHM) vs. Imagery Fists or Feet Movement (IFM): IHM vs. IFM;

### 2.1. Pre-Processing

The Dataset was imported into the EEGLAB v2019.1, where the signal of the three repetitions of each task and each subject were concatenated on one signal and decomposed into its main components using fastICA [38].

Three different techniques were applied and tested to improve the dataset’s Signal to Noise Ratio (SNR) and to eliminate non-cerebral signals, i.e., eye movements, environmental and channel noise. The first technique is simply based on applying a bandpass filter [1–48 Hz], while the second technique relies on the auto selection and exclusion of the EEG noisy components using SASICA software [22] on the filtered data. Finally, the last technique used a semi-automatic ICA (saICA) based procedure [16,18] to identify and classify artefactual components, where checking was applied to ensure no under or over-cleaning was reached by the saICA after filtering the data as for the previous method. 

### 2.2. Channels Selection

**Figure 1:**
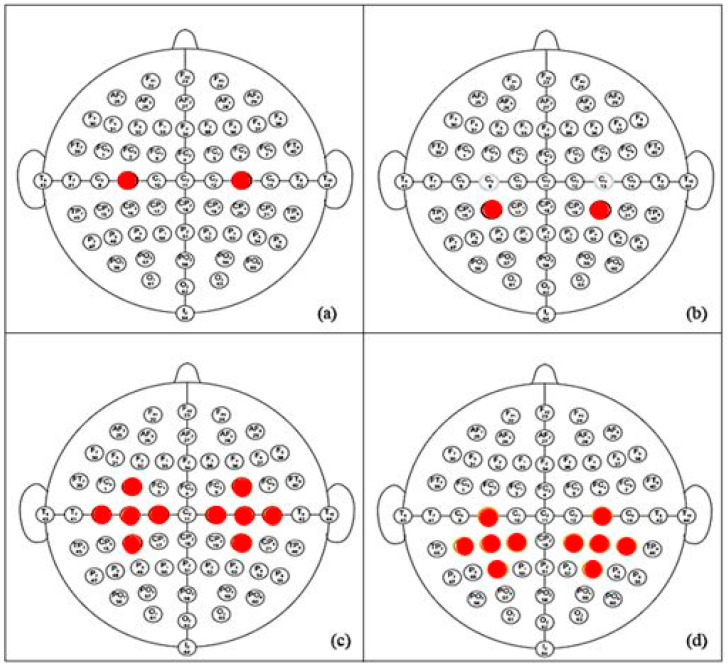
Different channels configuration tested: the red filled circles are located on the selected electrodes in the different configurations (**a**) C3/C4, (**b**) CP3/CP4, (**c**) ROI include C3/C4, and (**d**) ROI include CP3/CP4.

In order to detect the optimal groups of channels that give the best performance of the model, various classification processes have been performed with different pre-classification selected group of channels commonly used because placed on the region of motor cortex area [39]:

- C3/C4: using only the data from C3 and C4 channels.
- CP3/CP4: using only the data from CP3 and CP4 channels.
- ROIC3/ROIC4: includes FC3, C5, C3, C1, CP3, FC4, C2, C4, C6, CP4.
- ROICp3/ROICp4: includes C3, CP5, CP3, CP1, P3, CP2, C4, CP6, P4 as is shown in Figure 1a–d, respectively.

### 2.3. Features Extraction

Event-Related Desynchronisation [27,40] in alpha, beta, and alpha plus beta was applied to the data (filter, SASICA, and saICA).

Three finite impulse response filters were used to compute the ERD in three different frequency bands and for each EEG channel, ERD_A (i.e., the desynchronisation value obtained integrating the alpha band between 8–13 Hz), ERD_B (i.e., the desynchronisation value obtained integrating the beta band between 14–25 Hz), and ERD_AB (i.e., the desynchronisation value obtained integrating the alpha lus band between 8–25 Hz). 

ERD [27] was calculated using a pre-stimulus ‘Rest’ of 2 s (i.e., two seconds before the onset stimulus) and 2 s post-stimulus ‘Task’ (i.e., two seconds after the onset stimulus) across all trials and subjects. Once the two PSD (on the Rest and on the Task) were calculated, the ERD was abstained by the following formulation:

$$\mathrm{ERD}=\frac{\sum_{band}PSD\left(Task\right)-\sum_{band}PSD\left(Rest\right)}{\sum_{band}PSD\left(Rest\right)}$$

‘band’ subscript refers to alpha, beta and alpha plus beta.

### 2.4. Classification

The performance of three different classifiers was investigated, namely SVM, KNN, and TREE. SVM [41] is a supervised machine learning model that uses classification algorithms for two-group classification problems [42]. The second classifier is KNN [43], representing a non-parametric machine learning method where the input consists of the k closest training examples in feature space, while the output depends on whether KNN is used for classification or regression [44]. Finally, TREE classifier [45] was used as a predictive modelling approach. It uses a decision tree (as a predictive model) to go from observations about an item (represented in the branches) to conclusions about the target value of the item (represented in the leaves) [46].

The hyperparameters of each classifier have been optimised in a way that minimises five-fold cross-validation loss by using Bayesian optimisation [47].

### 2.5. Optimisation Channels Selection

A new optimisation method has been proposed in this study for data-driven channel selection. In contrast with the previous channel combinations which have been selected and fixed before the classification procedure [39] an optimisation system has been created to select the optimal combination of channels that gives the best model for each task.

This optimisation system initially works by computing the weight of each feature by using the relieff Matlab function, which calculates the Rank importance of predictors [34].

The descending order of the features based on their weight was given, then 64 groups of features were created, where the first group includes only the first feature in the feature order vector, while the second group includes the first and second features in the features order vector, and so on until the last group which includes all the features.

**Figure 2:**
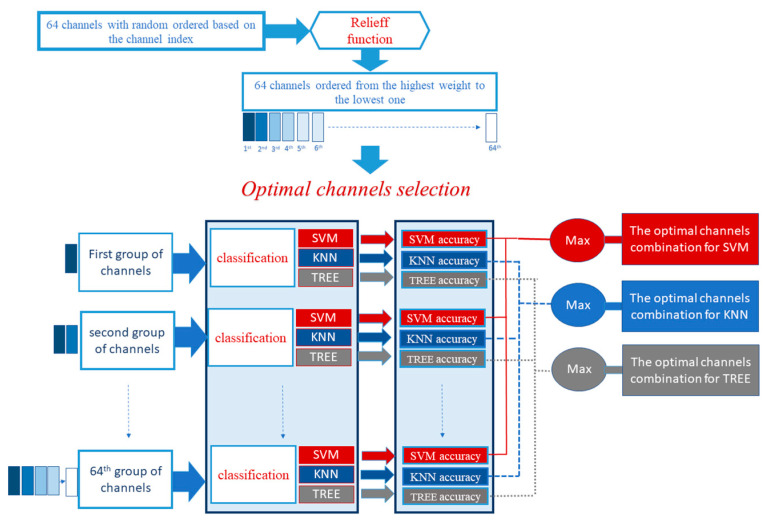
Schematic representation of the channel optimisation process and classification.

Later on, a 64-classification procedure will be performed using the 64 features group combination, and the combination of the features that give the highest accuracy was selected as the optimal group of channels. The previous procedure is explained in Figure 2.

## 3. Results

### 3.1. Comparison between the Cleaning Methods

Three different cleaning techniques (Filter, SASICA, saICA) were applied separately on the EEG signal. Then the effect of the cleaning technique on the accuracy of the pattern classification system was calculated and tested by creating all the possible combinations with features (ERD_A, ERD_B, ERD_AB), classifiers, and channels selection technique proposed in this study. The accuracy was calculated following the formula:

$$Accuracy=\frac{\left(\mathrm{TP}+\mathrm{TN}\right)}{\left(\mathrm{TP}+\mathrm{TN}+\mathrm{FP}+\mathrm{FN}\right)}$$

where TP indicates the True Positive, TN the True Negative, FP the False Positive and FN the False Negative. The accuracy is obtained as the proportion of correct predictions (both true positives and negatives among the total number of cases examined.

The results with the highest accuracy have been obtained using the ERD_AB as a feature, the SVM as a classifier, and the optimal channels selection technique.

A significant difference in the accuracy value has been noticed based on the cleaning techniques among the combination of the different tasks (RHM vs. IHM, RFM vs. IFM, RHM vs. RFM, IHM vs. IFM). 

The saICA technique showed the highest capability to clean the EEG signal without removing the important components. Thus, the data, cleaned with this method, give the highest accuracy across tasks comparison (mean = 94.25, SD = 3.86), followed by the data cleaned with SASICA (mean = 61.75, SD = 8.80), while the filter application performs the lowest accuracy (mean = 53, SD = 8.67). 

The obtained accuracy using the three different cleaning techniques is summarised in Table 1.

Thus, the results reported below from now on refer to the saICA cleaned data.

### 3.2. General Comparison across Methods

#### 3.2.1. An Overview across All the Proposed Techniques

Several IM and RM EEG pattern classification methods have been created with different features (ERD_A, ERD_B, ERD_AB), several channel groups (C3/C4, CP3/CP4, ROI C3/C4, ROI CP3/CP4, and optimal channels) and classifiers (SVM, KNN, TREE). The performance has been tested by calculating the accuracy among four different tasks combinations (RHM vs. IHM, RFM vs. IFM, RHM vs. RFM, IHM vs. IFM). Table 2 summarises the classification accuracy among the different techniques.

Detailed analysis for each test is presented below.

The best results were obtained using ERD_AB as a feature and SVM as a classifier and the combination of the optimal channel (RHM vs. IHM = 98%, RFM vs. IFM = 94%, RHM vs. RFM = 96%, and IHM vs. IFM = 91%). Extra analysis has been carried out in the following sections.

#### 3.2.2. Comparison between the Channel Selection Techniques

**Figure 3:**
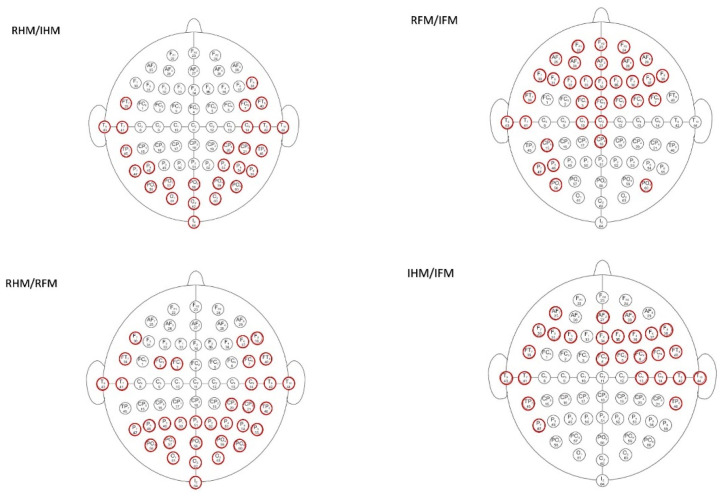
Optimal channel selection for a particular combination of parameters (ERD_AB as feature and SVM as a classifier) and among the four different combination tasks.

Across all the tasks combinations, it has been found that the optimal channel selection techniques return the highest accuracy (mean = 94.75, SD = 1.49%) followed by ROI C3/C4 (mean = 87.5, SD = 3.2%), ROI Cp3/Cp4 (mean = 80.75, SD = 4.13%), C3/C4 (mean = 76, SD = 13.47%) and the lowest values obtained using CP3/CP4 (mean = 69.75, SD = 7.22%). Figure 3 shows the optimal channel selection for a particular combination of parameters (ERD_AB as feature and SVM as a classifier) and among the four different tasks combinations (RHM vs. IHM, RFM vs. IFM, RHM vs. RFM, IHM vs. IFM). As shown in meta-analysis by [48], many studies show recruitment of frontoparietal and central areas during the execution of the motor imagery of the lower and upper limbs.

**Figure 4:**
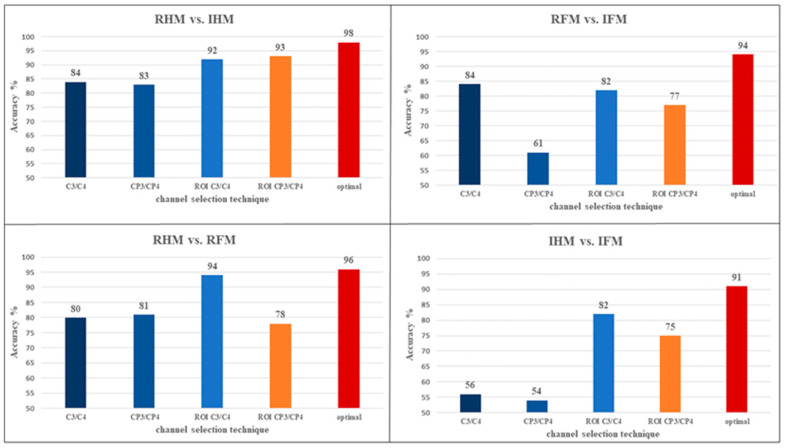
Column chart specifies the effect of the channel selection technique on the classification percentage accuracy to classify between different tasks. Showed that the highest accuracy is obtained by using the optimal channels selection technique. Using Semi-Auto ICA cleaned data and ERD_AB as a feature, SVM as a classifier.

The classification accuracy of different BCIs was calculated by using ERD_AB as a feature and SVM as a classifier, and different sets of channels (C3/C4, CP3/CP4, ROI C3/C4, ROI CP3/CP4, and optimal channels) to evaluate the performance of each group, as reported in Figure 4.

#### 3.2.3. Comparison between the Features

It has been found that the ERD_AB returns the highest accuracy across tasks comparisons (mean = 94.75 ± SD = 1.49%) followed by ERD_B (mean = 92.25 ± SD = 2.136%) and the lowest values obtained using ERD_A (mean = 88.25 ± SD = 1.25%).

**Figure 5:**
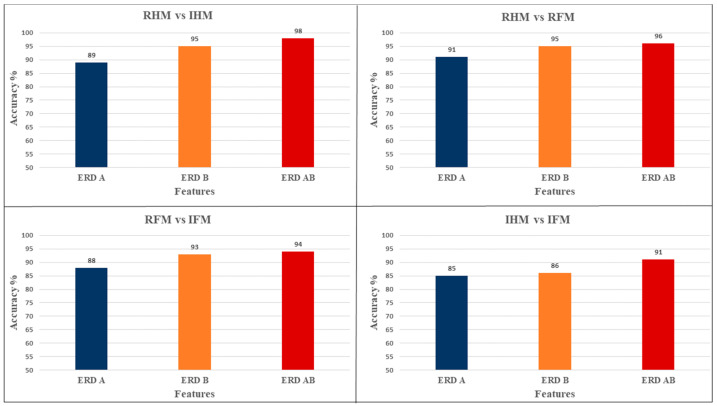
Column chart specifies the effect of the features on the classification percentage accuracy to classify between different tasks using semi-auto ICA cleaned data with the optimal channel selection and SVM as a classifier. Showed that the highest accuracy is obtained by using ERD_AB as a feature.

The classification accuracy of different BCIs was calculated by using the optimal channels selection and SVM as a classifier and different sets of features (ERD_A, ERD_B, and ERD_AB) in order to evaluate the performance of each group, as reported in Figure 5.

#### 3.2.4. Comparison between the Classifiers

It has been found that the SVM returns the highest accuracy among all the tasks combinations (mean = 94.75 ± SD = 1.49 %) followed by KNN (mean = 87.75, SD = 1.031 %) and the lowest values obtained using TREE (mean = 84.75, SD = 5.72%). This result is explained in Table 3 which summarises the classification results.

The SVM classifier has always provided the best mean accuracy with respect to different single features as reported in Table 3. All the three classifiers achieve classification accuracy above 60% even when the features selected belong only to ERD_A and ERD_B set of features, but a clear increase is recorded with the SVM performance with respect to KNN and even more respect to TREE classifier.

**Figure 6:**
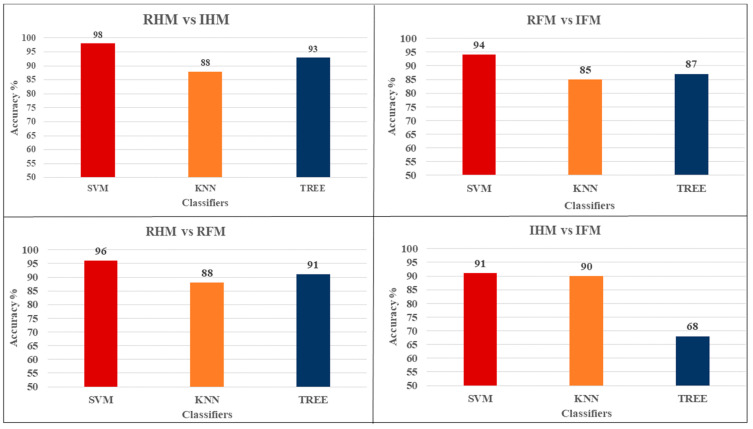
Column chart specifies the effect of the features on the classification accuracy to classify between different tasks using the optimal channels selection and ERD_AB as a feature. Showed that the highest accuracy is obtained by using the SVM classifier.

The classification accuracy of different BCIs was calculated by using the optimal channels selection and ERD_AB as a feature and different sets of classifiers (SVM, KNN, and TREE) in order to evaluate the performance of each group, as reported in Figure 6.

### 3.3. Evaluation of the Optimal Channel Selection Technique

In order to evaluate the performance of the proposed optimal channels selection technique (on ERD_AB as a feature and SVM as a classifier), several parameters (sensitivity, specificity, Area under the Curve (AUC), True Positive (TP), True Negative (TN), False Positive (FP), and False Negative (FN) have been calculated and summarised in Table 4.

The novel channels selection technique showed the highest fit with (RHM vs. IHM) task with Sensitivity = 97%, specificity = 99%, AUC = 0.99, TP= 194/200, TN = 199/200, FP = 1/200 and FN = 6/200.

## 4. Discussion and Conclusions

With the aim to define a pattern classification system for use in a BCI to classify between the real and the imagined movements, facilitating the interaction between people with limited motor abilities and their environment, a PhysioNet data set has been used [35], that includes EEG signals acquired during the performance of four different tasks: Real Hand Movement (RHM); Imagery Hand Movement (IHM); Real Fists or Feet Movement (RFM), and Imagery Fists or Feet Movement (IFM).

The goal of this study was to compare between different sets of pre-processing techniques (filtering, SASICA, and saICA), features (ERD in Alpha, Beta, and Alpha + Beta range), the different combinations of EEG channels (C3/C4, CP3/CP4, RoI centred on C3/C4 and RoI centred on CP3/CP4 and optimal channels combination), and different classifiers (SVM, KNN, and TREE) in order to evaluate their performance in a pattern classification system for use in a BCI.

The overall results have highlighted that the combination of the saICA as a cleaning method, the features ERD_AB, the optimal channel selection technique as the best channel configuration, and the SVM classifier give the best model solution, showing the highest accuracy values.

The different pre-processing approaches, based on filtering and independent component analysis methods, have been tested to reduce the signal-to-noise ratio. In this context, a semi-automated offline algorithm saICA [16,18] and a fully automated system SASICA [22] provided the best results with respect to the filtering data applying a pass-band filter followed by a notch filter.

The saICA outperforms the other methods because it controlled for over cleaning and down cleaning effects. In particular, while the filter approach probably suffers from an intrinsic down cleaning, keeping the motor artifacts that compromise the ERD feature extraction and consequently the classification, SASICA fully automatic version instead, over-cleaning the data, deletes some physiological activity during the task, compromising the feature extraction classification as well. 

Moreover, regarding the necessity of cleaning the data, different pre-processing techniques, referred to supervised ICA approach, were highly time-consuming. On this aspect, neural networks can help since they do not need a strong pre-processing as shown in some studies [49]. In those studies, only minimal pre-processing such as removing or interpolating bad channels were used and all the rest was left to the burden of learning from a potentially noisy signal to the neural network [49]. On the other hand, machine learning methods based on artificial neural networks with the ability to use techniques that allow a system to automatically detect and classify features from raw data, including unsupervised training of raw input data (i.e., automatic feature selection and dimensionality reduction), are highly computationally expensive to train and to determine the hyperparameters. Consequently, the choice of the hyperparameters is mainly user-dependent [49]. 

The analysed EEG signal features have been the event-related desynchronisation ERD and synchronisation (ERS) in the alpha [8–13 Hz], beta [14–25 Hz], and alpha plus beta [8,9,10,11,12,13,14,15,16,17,18,19,20,21,22,23,24,25] bands, highly suitable for motor execution and MI. The ERD is the acquisition recording method to evoke through the visual stimulation, linked with motor-related brain functions, mainly execution and imagery of motor actions [50]. Two different types of ERD features can be differentiated: one short-lasting, localised into occipital areas and involving upper alpha components which most likely reflects primary visual processing and feature extraction, and the other longer-lasting, more widespread, prominent over parietal areas and maximal for lower-alpha components, mostly related to cognitive processing and mechanisms of attention [50]. Alpha ERD selectivity was mainly observed at motor areas, whereas beta ERD responses selective to motion obeying this law were recorded at motor and prefrontal nonmotor sites. Unilateral voluntary upper limb movement is accompanied by an ERD feature in the alpha and beta bands localised over the contralateral sensorimotor area [27]. The results related to ERD features within both frequency bands (alpha plus beta) were consistently stronger, arose faster, and more widespread while observing motion [51]. In our case, the best results obtained on alpha plus beta ERD support that both bands are involved during motor imagery but also during motor action since the best accuracy classification was obtained in those bands when considered together with respect to the only alpha or beta bands.

The test on different combinations of channels to detect the optimal groups of features returned the highest accuracy [52] on the classification percentage between the different tasks for the optimal channel selection techniques, with a mean of 94.75% accuracy applying the SVM classifier. It provided the best results with respect to single-channel selection, using only the data provided by channels C3 and C4 and CP3 and CP4, and ROI C3/C4 and ROI CP3/CP4. Using a single-channel approach might be a solution to avoid computational time according to [17]. In our opinion, the single-channel solution presents some issues in all the cases the task under investigation involves a long-range and distributed brain network [53]. Since the MI signal derives its activities from a distributed network [54], picking a single channel or an average of channels based on a topographic map could be misleading, especially if the aim is to describe the whole neuronal communication system [49,52,53]. Specifically, when an electrical potential is generated by a neuronal group, its activity is not only recorded from the electrode closest to this source but also from distant ones, due to the electric field propagation phenomenon. Consequently, each channel on the scalp derives its signal from more than one source. This problem worsens with the increasing number of sources activated at the same time. The generators of EEG activity cannot be reliably inferred based on a priori selected single channels, or a limited group of channels, due to the electric/magnetic field propagation problem. Moreover, using information coming from only one electrode can be misleading especially when the activated network is spread among the entire scalp [17,52]. In this regard, methods able to extract the under-investigated neural source by combining the activity from all the electrodes, are suitable for overcoming possible misleading results by avoiding the choice of a single electrode or averaging a group of electrodes. This should be the reason why the optimal channel selection has given the highest accuracy.

In addition, in the present study, the performance of three different classifiers SVM, KNN, and TREE were tested and compared at (i) the single feature set selected, (ii) concerning the channel group adopted, and (iii) for each of the four tasks analysed. After the optimisation of the hyperparameters for each classifier using the Bayesian optimisation approach [55] the best results were provided by the SVM classifier:

(i) even if all the three classifiers achieve classification accuracy above 60%, a clear increase is recorded with the SVM also considering only ERD_A and ERD_B features (see Table 3);
(ii) with respect to the channel group adopted SVM classifier rises always the higher mean value (see Table 5);
(iii) among all the tasks combination (mean= 94.75 ± SD = 1.49%). For each of the analysed single tasks and among the different combinations of channels group the SVM classifier has always provided the best mean accuracy with respect to different single features as reported in Table 2. Our results are in line with other literature on MI in BCI that support SVM as the best and most used classifier in this context [56,57].

The aim of pooling features and testing these different combinations of task conditions, channels group, and classifiers was to investigate how their combination might improve the classification accuracy for a reliable MI-based BCI.

Finally, some consideration among the tasks classifying between RHM and IHM gives the highest accuracy 98%, while comparing between IHM and IFM give the lowest accuracy 91% by using the same features and the same manner to select the channels (see Table 1). The better performance obtained compared between imagery tasks still represents a challenge, probably due to the higher variability between the performances among the volunteers and in particular, due to the absence of closed-loop feedback that might test the accuracy of the task performed by the volunteers. 

To the best of our knowledge, we provide better overall classification results by comparing the results obtained with other studies working with the same data sets [58,59,60,61]. In particular, in Roots et al. [58], the 83.8% accuracy was obtained between the imagery movement task with the EEGNet Fusion, while in the proposed approach using ERD_AB and the optimal channels combinations with SVM classifier we reached 91% of the accuracy in the same task.

In other studies [59,60,61], that have used deep learning CNN (Convolutional Neural Network) to classify between events in IHM vs. IFM, obtained, respectively, 96%, 86%, and 85.9% of accuracy but selecting only 10 subjects. The results presented in this study reached, instead, 91% of accuracy using all the subjects by applying the SVM as a classifier. Based on different time-domain features and classifiers but the same observed task, Alomari and colleagues [62] used Movement-Related Cortical Potentials (MRCP) and Neural Networks (NN) to classify between real and imagined hand movement have obtained an accuracy of 89% but involving only the first 6 subjects. Once again, in our study by using all the 100 subjects and combining ERD_AB as a feature and SVM classifier we raised accuracy equal to 98% by using the same tasks. Differently, Sleight and colleagues [63], used EEG averaged power and SVM to classify between RHM and IHM and they got accuracy equal to 69% with respect to our 98% of accuracy. 

From the channels’ combination point of view, this study proposed a new method to obtain the combination of the optimal channels. This method was able to obtain the highest accuracy compared to fixed channels selection. However, in some specific BCI cases, the lowest number of channels is mandatory and preferred. The RoI and the selected channels configurations are preferable related to physiological hypotheses, while multiple channels selected by data-driven approaches might provide increased accuracy results but less accurate from a physiological and anatomical perspective. For this reason, the number of channels and the way to select them might be a good compromise to be decided on the BCI application purpose. 

Finally, from a computation point of view, the proposed model represents huge improvements in testing time, only 5 ms per sample, compared to the 107 ms when is used a convolutional neural network [58]. Moreover, even for the training time, this model requires just around 60 min to calculate the combination of the optimal channel for each feature and each classifier and to train the model against the days needed for training neural networks.

In conclusion, in this paper, we have presented a study based on data from the PhysioNet database. The automatically data-driven channel selection combined with an SVM classifier and a saICA as a pre-processing method has increased the classification accuracy for motor and imagery movement tasks to other approaches already presented in the literature.

## 5. Open Challenges

As a conclusion of our work, we provide a list of open challenges:

1. The use of different features such as Common Spatial Patterns (CSP) compared to ERD/ERS might be interesting to be tested.
2. The application of different feature ranking approaches might improve the accuracy and simultaneously lower the number of channels selected.
3. Different feature selection methods such as the Akaike Information Criterion (AIC) might guide the users to consider an optimal number of EEG channels.
4. Testing the accuracy of the single-subject performance instead of the accuracy at the group level.

## Figures and Tables

**Table 1:** Comparing the accuracy of classification using three different cleaning techniques (Filter, SASICA, and saICA) using ERD_AB as a feature, the optimal channels selection, and SVM classifier.

	RHM vs. IHM (%)	RFM vs. IFM (%)	RHM vs. RFM (%)	IHM vs. IFM (%)	Mean ± Standard Deviation
Filter	45	61	60	46	53± 8.67
SASICA	68	50	60	69	61.75 ± 8.80
saICA	98	94	96	91	94.25 ±3.86

**Table 2:** Summarise the classification accuracy using different sets of features (ERD_A, ERD_B, and ERD_AB) different sets of channel selection methods (C3/C4, CP3/CP4, ROI C3/C4, ROI CP3/CP4, and optimal channels) and different sets of classifiers (SVM, KNN, and TREE) to classify between different tasks combinations (RHM vs. IHM, RFM vs. IFM, RHM vs. RFM, and IHM vs. IFM).

Channel Selection	Features	RHM vs. IHM (%)			RFM vs. IFM (%)			RHM vs. RFM (%)			IHM vs. IFM (%)		
		SVM	KNN	TREE	SVM	KNN	TREE	SVM	KNN	TREE	SVM	KNN	TREE
C3/C4	ERD_A	50	51	52	59	57	55	62	61	60	56	57	54
	ERD_B	72	72	70	61	63	62	65	63	58	58	60	56
	ERD_AB	84	81	84	84	82	81	80	78	77	56	55	55
CP3/CP4	ERD_A	56	54	52	58	56	58	62	60	58	57	55	57
	ERD_B	70	70	68	65	67	65	68	65	65	54	55	55
	ERD_AB	83	82	80	61	60	60	81	78	75	54	54	55
ROI C3/C4	ERD_A	78	81	61	83	79	61	87	88	67	79	68	60
	ERD_B	92	78	74	86	77	69	96	82	78	75	68	58
	ERD_AB	92	89	86	82	79	66	94	87	83	82	73	60
ROI CP3/CP4	ERD_A	69	63	56	75	66	56	79	71	62	67	66	56
	ERD_B	86	75	74	79	66	68	91	87	73	65	65	60
	ERD_AB	93	90	82	77	71	61	78	77	60	75	71	68
Optimal channels	ERD_A	89	80	73	88	76	69	91	89	72	85	75	69
	ERD_B	95	85	84	93	82	76	95	90	83	86	70	65
	ERD_AB	98	88	93	94	85	87	96	88	91	91	90	68

**Table 3:** Classifiers mean accuracy among channels selection techniques, with respect to the features over the four tasks combinations.

Channel Groups	Features	RHM vs. IHM (%)			RFM vs. IFM (%)			RHM vs. RFM (%)			IHM vs. IFM (%)		
		SVM	KNN	TREE	SVM	KNN	TREE	SVM	KNN	TREE	SVM	KNN	TREE
CP3/CP4, ROI C3/C4, ROI CP3/CP4, Optimal channels	ERD_A	68	66	59	73	67	60	76	74	64	69	64	59
	ERD_B	83	76	74	77	71	68	83	77	71	68	64	59
	ERD_AB	90	86	85	80	75	71	86	82	77	72	69	61

**Table 4:** Summarising the results obtained for the optimal channels selection technique showing the results obtained for the sensitivity, specificity, area under the curve AUC, true positive (TP), true negative (TN), false positive (FP) and false negative (FN) of a pattern classification system for use in a BCI using the semi-auto cleaned ICA data with the ERD_AB as a feature and SVM as a classifier.

	RHM vs. IHM	RFM vs. IFM	RHM vs. RFM	IHM vs. IFM
Accuracy	98%	94%	96%	91%
Sensitivity	97%	92%	91%	87%
Specificity	99%	90%	99%	86%
AUC	0.99	0.96	0.97	0.93
TP	194/200	184/200	181/200	174/200
TN	199/200	181/200	197/200	171/200
FP	1/200	19/200	3/200	29/200
FN	6/200	16/200	19/200	26/200

**Table 5:** Classifiers mean accuracy among tasks and selected features, with respect to channels selection techniques.

Features	Channel Groups	RHM vs. IHM (%), RFM vs. IFM (%), RHM vs. RFM (%), IHM vs. IFM (%)		
		SVM	KNN	TREE
ERD_A ERD_B ERD_AB	C3/C4	65.58	65	63.66
	CP3/CP4	64.08	63	62
	ROI C3/C4	85.5	79	68.58
	ROI CP3/CP4	77.8	72	64
	Optimal channels	91.75	83.16	77.5

## Data Availability

The study did not report any data.
